# Variants in FAT1 and COL9A1 genes in male population with or without substance use to assess the risk factors for oral malignancy

**DOI:** 10.1371/journal.pone.0210901

**Published:** 2019-01-18

**Authors:** Chia-Min Chung, Chung-Chieh Hung, Chien-Hung Lee, Chi-Pin Lee, Ka-Wo Lee, Mu-Kuan Chen, Kun-Tu Yeh, Ying-Chin Ko

**Affiliations:** 1 Environment-Omics-Disease Research Center, China Medical University Hospital, Taichung, Taiwan; 2 Graduate Institute of Biomedical Science, China Medical University, Taichung, Taiwan; 3 Department of Psychiatry, China Medical University Hospital, Taichung, Taiwan; 4 Department of Public Health, College of Health Sciences, Kaohsiung Medical University, Kaohsiung, Taiwan; 5 Department of Otolaryngology, Kaohsiung Medical University Hospital, Kaohsiung, Taiwan; 6 Oral Cancer Center, Changhua Christian Hospital, Changhua, Taiwan; 7 Department of Pathology, Changhua Christian Hospital, Changhua, Taiwan; Charles P. Darby Children's Research Institute, UNITED STATES

## Abstract

A number of genetic variants were suggested to be associated with oral malignancy, few variants can be replicated. The aim of this study was to identify significant variants that enhanced personal risk prediction for oral malignancy. A total of 360 patients diagnosed with oral squamous cell carcinoma, 486 controls and 17 newly diagnosed patients with OPMD including leukoplakia or oral submucous fibrosis were recruited. Fifteen tagSNPs which were derived from somatic mutations were genotyped and examined in associations with the occurrence of oral malignancy. Environmental variables along with the SNPs data were used to developed risk predictive models for oral malignancy occurrence. The stepwise model analysis was conducted to fit the best model in an economically efficient way. Two tagSNPs, rs28647489 in *FAT1* gene and rs550675 in *COL9A1* gene, were significantly associated with the risk of oral malignancy. The sensitivity and specificity were 85.7% and 85.5%, respectively (area under the receiver operating characteristic curve (AUC) was 0.91) for predicting oral squamous cell carcinoma occurrence with the combined genetic variants, betel-quid, alcohol and age. The AUC for OPMD was only 0.69. The predictive probability of squamous cell carcinoma occurrence for genetic risk score without substance use increased from 10% up to 43%; with substance use increased from 73% up to 92%. Genetic variants with or without substance use may enhance risk prediction for oral malignancy occurrence in male population. The prediction model may be useful as a clinical index for oral malignancy occurrence and its risk assessments.

## Introduction

Oral squamous cell carcinoma (OSCC) is a growing public health problem in the world[1]. Various anatomical sites of oral cavity showed different incidence rates of OSCC and their different treatment strategies, with either single treatment or a combination of surgery, radiotherapy and chemotherapy[2]. The disease continues to have a poor prognosis with a 5-year survival rate of <50%[3]. The survival rate increased with early detection which suggests the importance of the early prevention for OSCC in reducing morbidity and mortality [4].

With increased understanding of genetics and environmental risk factors in oral tumorigenesis, novel approaches have developed for prevention, early detection, risk stratification and treatment of OSCC. Major risk factors associated with the occurrence of oral malignancy are genetic risk factors [5, 6] and environmental risk factors, including betel quid (BQ) chewing and cigarette and alcohol consumption [7, 8]. Genetic and environmental risk factors have interactive effects on the occurrence of oral malignancy [9].

Oral potentially malignant disorders (OPMD) are early clinical features that are thought to undergo histopathological and molecular changes resulted in invasive oral cancer [10, 11]. OPMD can be visually detected in the oral cavity and it is a well-established pre-cancer stage. OPMD like OSCC occurrence is primarily caused by risk exposures such as tobacco smoking, betel quid chewing and alcohol [12, 13]. Because inter-individual and inter-population differences in risk [11] could be partially explained by different distributions of genetic variants, personal variation in the ability to metabolize carcinogens and effective DNA repair of the damage may be caused by genetic factors [14, 15]. Identifying genetic factors that render individuals susceptible to OPMD risk could have practical significance in terms of identifying potential biomarkers for earlier identification of OSCC [16]

Genetics is an important risk factor for oral malignancy occurrence [5, 6], but whether genetic information could improve the prediction of OSCC occurrence risk remains unclear. Therefore, we identified the significant variants and developed a model that integrates genetic profiling and environmental factors to detect high-risk groups for oral malignancy occurrence. Since OPMDs require long-term monitoring to assess their risk of developing OSCC, the prediction model may be useful in the identification of different risk groups to suggest intervention and would be increasingly important in reducing risks of OSCC.

## Material and methods

### Study population

A total of 360 newly diagnosed patients with OSCC and 486 controls were recruited from the Department of Dentistry and the Department of Otorhinolaryngology, Kaohsiung Medical University Hospital (KMUH) in Southern Taiwan and Changhua Christian Hospital (CCH) in mid-Taiwan. The control samples included patients with eye problems (cataract and glaucoma), bone fractures, and subjects undergoing physical checkups from KMUH and CCH. Another 161 subjects composed of 17 newly diagnosed patients with OPMD including leukoplakia or oral submucous fibrosis and 144 subjects without oral malignancy were recruited from a program for cancer screening by China Medical University Hospital (CMUH). Cancer screening program is a community-based program for substance users to detect oral potentially malignant disorders and oral cancer early. Data on social–demographic factors, anthropometric parameters, cigarette smoking, alcohol drinking and BQ chewing habits, medical history, and current medications were obtained by interviewing the subjects. BQ chewers were the subjects who had consumed at least one quid of any type of betel or areca nut product per day for a minimum of 6 months in their lifetime, and current BQ chewers were defined as participants who had chewed these products within the year prior to the interview. Substance use of alcohol and cigarettes were defined with the same criteria of BQ use. An individual without substance use of alcohol, BQ, and cigarettes was defined as the nondrinker, nonchewer, and nonsmoker, respectively. Detailed patterns of BQ, alcohol, and tobacco use comprised of addicted types, age at initial use, daily consumption, frequency of usuage, years of substance use and achievement of abstinence were described in the previous paper [17]. This study was approved by the Institutional Review Boards of CMUH, KMUH and CCH, informed consent committee on human subjects, and biospecimen unitization committee.

### Selection of tag SNPs of susceptibility candidate genes

Although a number of genetic variants were suggested to be associated with OSCC by genome-wide association studies, few variants are able to be replicated among the different population. Thus, attention has turned to the somatic mutations that are reported in the Cancer Genome Atlas that has shown promising genes associated with the initiation and progression of OSCC [18]. We evaluated and choose promising driver genes related to OSCC were nominated based on Cancer Genome Atlas and mutation rate greater than 20% by next-generation sequencing approaches [9, 19–21]. These genes included *TP53*, *CASP8*, *FAT1*, *NOTCH1* and *COL9A1*. Variants associated with cancer occurrence may be related to the acquired somatic mutations that drive cancer development. Based on linkage disequilibrium patterns of Han Chinese, Fifteen SNPs nearby somatic mutations among 5 genes with a minor allele frequency greater than 5% were selected from the HapMap database. SNPs were genotyped using Sequenom Mass ARRAY System (Sequenom, San Diego, CA, USA) at the Academia Sinica National Genotyping Centre (Taipei, Taiwan). The significant SNPs associated with OSCC were further genotyped and examined in association with subjects recruited from OPMD screening program. With the expectation to reduce numbers of variables to minimal and to reach high predictive power in the statistical models, we conducted stepwise model analysis to select risk factors to fit in the best model.

### Statistical analysis

Statistical analysis in this study was performed by using SAS 9.2 software. All genotype frequencies of control population were tested for Hardy–Weinberg equilibrium. The difference between the practical and expected number of each genotype was compared by the χ^2^ test. Hardy–Weinberg equilibrium was assumed for *P* value more than 0.05. Student *t*-test was applied to compare the age difference, whereas Pearson χ^2^ test or Fisher exact test was used to determining the difference of gender distribution and SNP genotype frequencies between case and control subjects. The false discovery rate (FDR) method was used for multiple testing corrections in genetic association studies. Logistic regression analysis [22] was performed to estimate the association between SNPs and OSCC. All tests are two-tailed, and a *P* value of <0.05 is considered to be statistically significant. The OR and the corresponding 95% confidence intervals (95%CI) were assessed by logistic regression. We calculated genetic risk scores (GRS) using risk allele frequencies, assuming independence of additive risks. Genetic risk scores were calculated from *FAT1* and *COL9A1*. A score of 1 was given to each T allele of COL9A1- rs550675 and G allele of FAT1- rs28647489. A multinomial logistic regression model including GRS and BQ use interaction term and adjusted for covariates was applied to examine in an association between OSCC risk and genetic variants. We employed ROC curves to compare the diagnostic ability of OSCC occurrence. Due to genetic influences on lifespan, we used age as follow-up years. Risk scores for oral malignancy were developed by Cox proportional hazard models.

## Results

The proportions of males were 95.3% in the OSCC case group, 97.4% in the control group (*P* = 0.1037). Percentage of gender was not significant. The proportions of participants were 100% males in the OPMD screening group. The mean age of the OSCC cases and normal control was 54.2 years (SD, 10.5), 51.7 and years (SD, 13.4) respectively (*P* = 0.01). The mean age was not significant between OPMD and normal group (age = 48.71(9.31), 47.12(12.79); p = 0.7432).

There were 86.9% the habit of smoked cigarettes in the OSCC groups, and 51% in the control groups (all *P* < 0.0001). Of the OSCC cases, 80.6% had the habit of consuming betel quid, 86.9% had smoked cigarettes and 68.1% had drinking alcohol. Of the control group, 13.4% had the habit of consuming betel quid, 51% had smoked cigarettes and 26.9% had drinking alcohol (*P* < 0.0001; Table 1). The distribution for a percentage of substance use in OPMD screening program was similar with OSCC group. Substance use of BQ, alcohol and cigarettes were not significant between OPMD and normal group (Right panel of Table 1).

**Table 1:** Comparing characteristics of the study subjects.

	Case-control study			Cancer screening program*		
variable	OSCC	Control		OPMD	Normal	
	N = 360	N = 486	P-value	(N = 17)	N = 144	P-value
Male, N (%)	343(95.3)	475(97.4)	0.1037	17(100)	159(100)	1
Age, year (SD)	54.2(10.2)	51.7(13.4)	< .0001	48.17(9.31)	47.12(12.78)	0.7432
	
Cigarette, N (%)						
None	47(13.1)	238(49)	< .0001	2(11.76)	15(10.42)	0.2425
Yes	313(86.9)	248(51)		15(88.24)	129(89.58)	
Alcohol, N (%)						
None	115(31.9)	335(73.1)	< .0001	5(29.41)	63(43.75)	0.3073
Yes	245(68.1)	131(26.9)		12(70.59)	81(56.25)	
BQ Chewing, N (%)						
None	70(19.4)	421(86.6)	< .0001	2(11.76)	39(27.08)	0.1704
Yes	290(80.6)	65(13.4)		15(88.24)	105(72.92)	

* Cancer screen program is a community-base program for substance users to detect oral potentially malignant disorders and oral cancer early.

Table 2 and S1 Table listed the genotype and allele frequencies of individual SNP in OSCC cases and control subjects. A total of 15 SNPs in 5 candidate genes were examined in associations with the occurrence of OSCC. We found that two SNPs, rs28647489 in *FAT1* gene and rs550675 in *COL9A1* gene, were associated with a higher risk of OSCC. Compared with those carrying wild-type genotype and allele, patients carrying variant GA and GG genotype of rs28647489 had an increased risk of OSCC (OR = 2.06; 95% CI, 1.05–4.05; OR = 2.85; 95% CI, 1.19–6.84). The CT and TT genotypes of rs550675 had the additive effects on the risk of OSCC (OR = 1.31; 95% CI, 1.01–1.65; OR = 2.63; 95% CI. 1.47–4.72; Table 2).

**Table 2:** Association between selected SNP derived from somatic mutations and the risk of OSCC occurrence.

Gene	SNP	Genotypes	Case	Control	P-value	FDR^&^	OR	95% CI	OR*	95% CI
			n(%)	n(%)						
TP53	rs11652704	**C/C**	267(75.0)	364(74.9)	0.8619	0.9313	1			
		C/T	82(23.0)	116(23.8)			1.52	(0.48–4.85)	1.4	(0.43–4.50)
		T/T	7(2.0)	6(1.3)			0.97	(0.69–1.36)	0.94	(0.66–1.32)
TP53	rs12951053	A/A	153(43.0)	212(44.1)	0.84683	0.9313	1			
		C/A	173(48.6)	219(45.5)			1.11	(0.61–2.03)	1.1	(0.82–1.46)
		C/C	30(8.4)	50(10.4)			1.41	(0.48–4.09	0.83	(0.51–1.37)
TP53	rs17882227	C/C	30(8.4)	45(9.3)	0.82826	0.9313	1			
		T/C	169(47.5)	217(44.7)			0.93	(0.28–3.06	1.17	(0.69–1.99)
		T/T	157(44.1)	224(46.0)			0.58	(0.18–1.95)	1.06	(0.62–1.80)
CASP8	rs6745051	A/A	190(53.4)	269(56.3)	0.30283	0.9313	1			
		C/A	133(37.4)	173(36.2)			1.25	(0.68–2.29)	1.09	(0.81–1.46)
		C/C	33(9.3)	36(7.5)			2.61	(0.74–9.22)	1.3	(0.78–2.16)
CASP8	rs7608692	A/A	17(5.0)	16(3.5)	0.77023	0.9313	1			
		A/G	139(40.5)	193(42.2)			0.42	(0.12–1.50	0.68	(0.33–1.39)
		G/G	187(54.5)	248(54.3)			0.51	(0.15–1.77)	0.71	(0.35–1.44)
CASP8	rs6754084	C/C	181(50.8)	243(50.0)	0.931	0.931	1			
		T/C	145(40.7)	204(42.0)			1.16	(0.64–2.10)	0.95	(0.71–1.29)
		T/T	30(8.4)	39(8.0)			0.91	(0.30–2.73)	1.04	(0.60–1.78)
FAT1	rs28647489	A/A	114(31.7)	193(39.7)	0.00569	0.0353	1			
		G/A	171(47.5)	219(45.1)			2.06	(1.05–4.05)	1.32	(1.01–1.80)
		G/G	75(20.8)	74(15.2)			2.85	(1.19–6.84)	1.72	(1.16–2.55)
FAT1	rs2306990	C/C	63(17.8)	71(14.8)	0.88318	0.9313	1			
		C/T	162(45.6)	244(50.8)			0.77	(0.31–1.93)	0.75	(0.51–1.11)
		T/T	130(36.6)	165(34.4)			1.63	(0.63–4.24)	0.89	(0.59–1.34
FAT1	rs11724817	A/A	90(25.2)	135(27.8)	0.92244	0.9313	1			
		A/T	175(49.0)	215(44.4)			1.88	(0.93–3.82)	1.22	(0.84–1.78)
		T/T	92(25.8)	135(27.8)			1.92	(0.87–4.27)	1.02	(0.67–1.56)
FAT1	rs2130909	C/C	112(31.4)	148(31.0)	0.56579	0.9313	1			
		T/C	170(47.8)	218(45.7)			0.84	(0.43–1.61)	0.75	(0.51–1.11)
		T/T	74(20.8)	111(23.3)			0.44	(0.19–0.99)	0.89	(0.59–1.34)
FAT1	rs10009030	A/A	62(17.6)	71(14.9)	0.31465	0.9313	1			
		C/A	178(50.4)	244(51.0)			0.55	(0.25–1.22)	0.84	(0.57–1.24)
		C/C	113(32.0)	163(34.1)			0.38	(0.16–0.93)	0.79	(0.52–1.20)
FAT1	rs2637777	G/G	149(41.8)	197(40.6)	0.56561	0.9313	1			
		G/T	154(43.3)	236(48.6)			0.56	(0.31–1.04)	0.85	(0.61–1.19)
		T/T	53(14.9)	52(10.8)			0.61	(0.22–1.69)	0.83	(0.56–1.22)
FAT1	rs10434309	C/C	115(32.3)	139(28.6)	0.544	0.9313	1			
		C/T	158(44.4)	225(46.4)	0.31928		1.14	(0.58–2.24)	0.86	(0.63–1.18)
		T/T	83(23.3)	122(25.0)			1.34	(0.61–2.95)	1.34	(0.84–2.13)
COL9A1	rs550675	C/C	156(43.3)	262(53.9)	< .0001	0.0002	1			
		C/T	136(37.8)	180(37.0)			1.27	(0.94–1.71	1.31	(1.01–1.65)
		T/T	68(18.9)	44(9.1)			2.6	(1.69–3.98)	2.63	(1.47–4.72)
NOTCH1	rs201174576	T/T	154(43.3)	207(43.2)	0.69211	0.9313	1			
		G/T	161(45.2)	208(43.4)			1.15	(0.62–2.10)	1.04	(0.78–1.40)
		G/G	41(11.5)	64(13.4)			0.53	(0.21–1.35)	0.86	(0.55–1.34)

* Statistics corresponding to logistic regression for association between risk of oral cancer and genetic SNPs after adjustment for age and gender.

^&^FDR: False discovery rate was used to control the error rate under multiple testing

We constructed GRS by using two significant SNPs in *FAT1* and *COL9A1* genes. The univariate associations of environmental risk factors, the use of alcohol, BQ, and cigarette, and GRS associated with OSCC risk are shown in Table 3. The GRS was associated with the risk of OSCC (unadjusted OR ranged from 1.64 to 4.86). After adjustment for age, and the use of alcohol, BQ, and a cigarette, the GRS remained independently associated with the risk of OSCC (OR ranged from 1.68 to 6.12).

**Fig 1:**
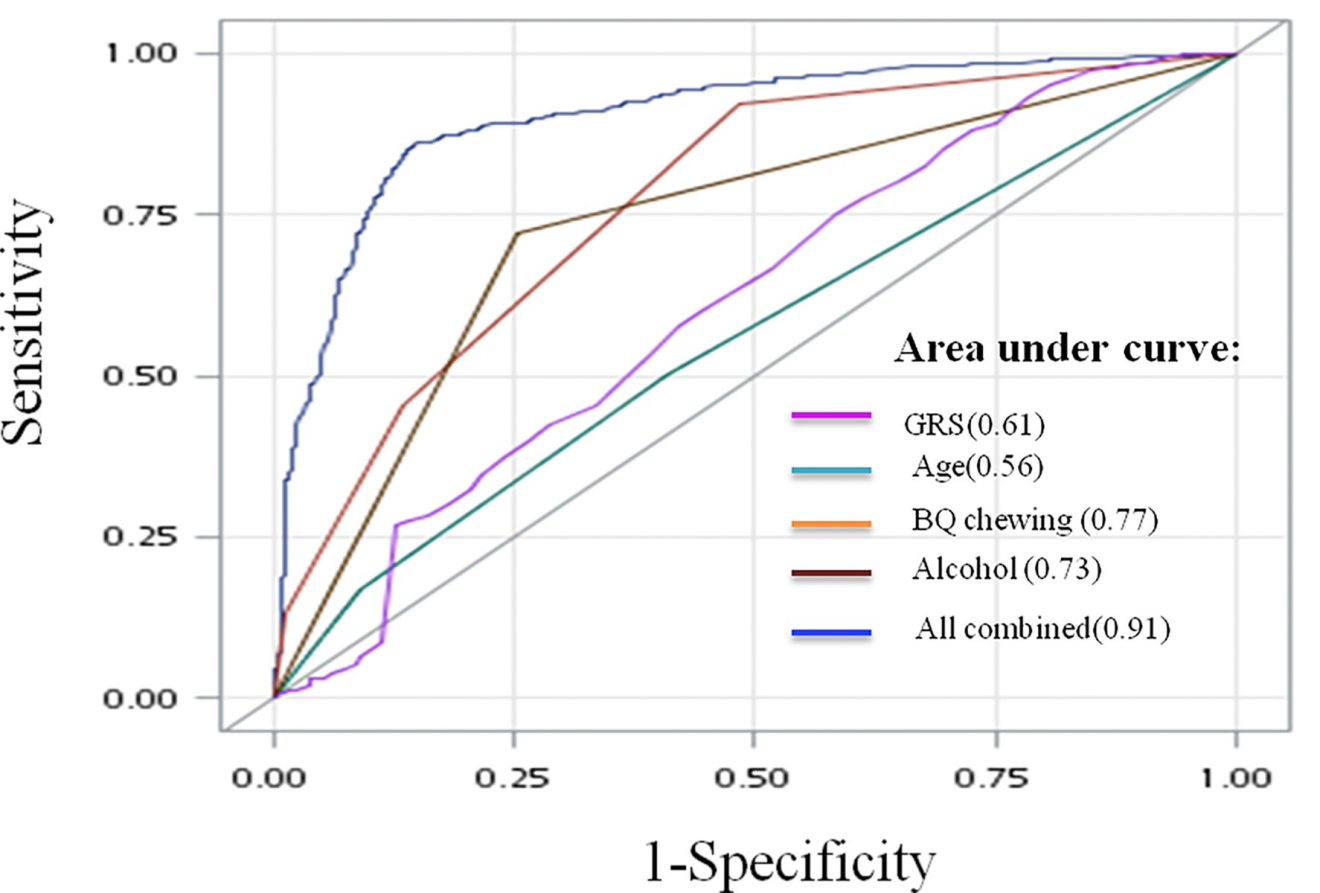
Receiver operating characteristic (ROC) curves and comparison of areas under the ROC curves for the GRS, age, alcohol and BQ use were used to predict OSCC occurrence.

Based on the results of Table 3, we conducted stepwise model analysis to select the significant factors for the best fit to prediction model of OSCC. The results were shown in the S2 Table. *FAT1*, *COL9A1*, BQ use and habit of consuming alcohol were included in the best fit model (p-value was set <0.05). The diagnostic ability for GRS and environmental factors was measured as areas under the ROC curves (AUC) in the prediction models. The AUC for GRS was 0.61. The AUC for BQ and alcohol were 0.77 and 0.73 respectively. The AUC of the combined genetic, BQ and alcohol were significant predictors for the occurrence of OSCC. The addition of the GRS into the age, BQ and alcohol use included model showed significant improvement in AUC (the AUC increased to 0.91) with 85.70% of sensitivity and 85.5% of specificity for OSCC occurrence, respectively (Fig 1).

**Table 3:** Predicted risks of environmental factors and genetic information for OSCC.

Parameters	OR(95% CI) ^b^	OR(95% CI) ^c^
Age	1.02(1.01–1.03)	1.03(1.01–1.05)
Alcohol	5.77(4.28–7.78)	1.85(1.23–2.77)
Smoking	6.39(4.49–9.11)	1.62(1.02–3.61)
Betel	26.83(18.55–38.82)	18.831(12.31–28.81)
FAT1- rs28647489		
A/A	Reference	Reference
G/A	1.32(0.97–1.8)	1.6(1.05–1.2.47)
G/G	1.72(1.16–2.55)	2.09(1.21–3.61)
COL9A1- rs550675		
C/C	Reference	Reference
T/C	1.27(0.94–1.71)	1.01(0.73–1.65)
T/T	2.60(1.69–3.98)	2.63(1.47–4.72)
Genetic risk scores ^a^		
0	Reference	Reference
1	1.64(1.13–2.38)	1.68(1.01–2.81)
2	4.86(1.76–13.40)	6.12(1.66–22.49)

^a^ Genetic risk scores were calculated from FAT1 and COL9A1. A score of 1 was given to each T allele of COL9A1- rs550675 and G allele of FAT1- rs28647489.

^b^ Statistics corresponding to logistic regression for association between risk of oral cancer and environmental factors and genetic risk scores in a single parameter model.

^c^ Statistics corresponding to logistic regression for association between risk of oral cancer and environmental factors and genetic risk scores in a multiple variables model.

We also conducted stepwise model analysis to select significant factors for the best- fit prediction model of OPMD. The results were shown in the S3 Table. The best fit models for OPMD screening were included FAT1, COL9A1, BQ use and habit of cigarettes smoking. The AUC for GRS was 0.61. The AUC for BQ and cigarettes were 0.58 and 0.51 respectively. The AUC of the combined genetic variants, BQ and cigarettes were 0.69.

According to AUC model, we calculated a predictive probability of OSCC occurrence for each group in term of genetic risk score BQ use and alcohol drinking. Predictive probability of OSSC occurrence increased with genetic risk score from 0.10 to 0.43 for the subject without substance use and 0.73 to 0.92 for the subject without substance use. Without genetic effects, the predictive probability of occurrence increased from 0.10 to 0.38 for BQ use and 0.10 to 0.29 for alcohol drinking respectively. The predictive probability of OSCC occurrence for genetic risk score with substance use increased from 73% to 92%. The synergic effects of the probability of occurrence for genetic risk score, BQ use and alcohol drinking increased from 0.10 to 0.92 (Table 4).

**Table 4:** Based on the genetic risk score BQ use and alcohol drinking, predictive probability of OSCC occurrence.

Genetic effects	BQ effects	Alcohol effects	Predictive probability of occurrence OSCC	95% CI	
**0**	**0**	**0**	0.10	0.06	0.16
**0**	**0**	**1**	0.29^#^	0.19	0.39
**0**	**1**	**0**	0.38^$^	0.33	0.47
**0**	**1**	**1**	0.73	0.64	0.82
**1**	**0**	**0**	0.20*	0.03	0.61
**1**	**0**	**1**	0.46	0.14	0.86
**1**	**1**	**0**	0.48	0.23	0.87
**1**	**1**	**1**	0.75^&^	0.52	0.97
**2**	**0**	**0**	0.43*	0.34	0.50
**2**	**0**	**1**	0.75	0.75	0.75
**2**	**1**	**0**	0.74	0.70	0.77
**2**	**1**	**1**	0.92^&^	0.91	0.94

^#^ Predictive probability of occurrence from alcohol drinking effects.

Alcohol drinking effects = (0.29–0.1)*100 = 19%;

^$^ Predictive probability of OSCC occurrence from BQ use effects.

BQ use effects = (0.38–0.1)*100 = 28%

Predictive probability of OSCC occurrence from genetic effects (GE) with/ without substance use.

*GE without substance use = (0.43–0.1)*100 = 33%

^&^GE with substance use (0.92–0.73)*100 = 19%

## Discussion

Although a number of genetic variants were suggested to be associated with OSCC occurrence by association studies [23, 24] and genome-wide association studies [25], however, few variants are able to be consistent in association with OSCC among the different population. Even variants in *P53* gene in meta-analysis comprised of 2298 OSCC and 2111 controls were not associated with OSCC occurrence [26]. Therefore, attention has turned to the somatic mutations identified by next-generation sequencing approaches which are reported in the Cancer Genome Atlas that has shown promising genes associated with the initiation and progression of OSCC [25, 27–29]. We found tagSNPs in the *FAT1* and *COL9A1* gene nearby somatic mutations that drive cancer development were associated with oral malignancy occurrence. COL9A1 encodes one of the three alpha chains of type IX collagen. The levels of methylation in *COL9A1* were decreased in breast tumor tissue [30] and variants associated with OSCC occurrence [9]. The FAT1 gene encodes a cadherin-like protein, which is able to potently suppress cancer cell growth [31, 32]. More recently, Morris et al. reported that FAT1 via mutation promotes Wnt signaling that drives the development of many types of human malignancy [33]. High mutation frequency in the FAT1 exclusively associated with HPV-negative head and neck squamous cell carcinoma (HNSCC) [18, 34]. Although a loss of function in *FAT1* were identified to be associated with HNSCC and OSCC in cell models, the frequencies of mutations were rare, suggesting that variants with large effect sizes will impact a small proportion of the population. It cannot be applied to screen general populations. Based on 1000 genome database, we identified two SNPs in *FAT1* and *COL9A1* gene that compared two major allelic frequency (A>G, SNP rs28647489 and C>T, SNP rrs550675) with other ethnic populations and found that were different from those of European, American, African, but the allelic frequency is similar with East Asia. These differences in allelic frequent distribution could be attributed to affect the risk of OC and OPMD occurrence in different ethnic populations. We showed that an interaction between additive genetic risk score of *FAT1* and *COL9A1* genes and BQ chewing has strong and graded associations with an occurrence of OSCC in case and control study and further confirmed that applied to early detection of OPMD risk.

Generally, OSCC is diagnosed in advanced stages, resulting in poor survival rates. Therefore, early oral malignancy prevention or detection are imperative where treatment in the pre-invasive stage offers the better prognosis and even the chance of cure. Using genetic and environmental factors to identify high‐risk individuals would be effective in reducing the incidence and mortality from OSCC. Chuang et al. reported that early detection OPMD led to a 21% reduction in stage III or IV oral cancer diagnosis and a 26% reduction in oral cancer mortality in cancer screening program targeting Taiwanese cigarette smokers, or betel quid chewers [35]. Although cancer screening program can reduce incidence and mortality of oral cancer, subject without substance use and low risk for OPMD who do not attend screenings program may decrease the effectiveness in reducing the incidence, mortality and survival rate of OSCC. We developed a new prediction model integrated with genetic information for improving the detectability of oral malignancy. We used two genetic variants for OPMD screening, with the least number of variants to achieve the highest economic efficiency and screening power. The predictive probability increased with genetic risk scores which ranged from 0.32 to 0.70 (S4 Table). Because of enhanced awareness in those with high genetic risk, our prediction model may assess high-risk individuals with/without substance use to improve early oral malignancy detection.

A linear equation was constructed to produce the risk scores (S3 Table and Table 3). Subjects were divided into three groups based on risk score and would assist in the screening of individuals in high risk and be helpful to the personalized prevention of OPMD. Apart from predicting the occurrence of OSCC, several risk models or risk scores have been built for risk assessment by using environmental factors, lifestyle and clinical data [36–39]. Our study integrated GRS and environmental factors to detect the risk of oral malignancy occurrence early. When this prediction is used for risk assessment in asymptomatic individuals, those who test at high-risk score may consider several preventive strategies. The most common strategy is increased surveillance which includes annual visual inspection by clinician and intervention in quitting of BQ use. Recently substance use, especially for BQ use is defined as an addictive substance by DSM-5 criteria that may provide the strategy in the cessation of BQ use [40]. The efficacy of prediction model may be useful in the identification of different risk groups to suggest intervention and would be increasingly important in the prevention and diagnosis of OSCC.

We integrated environmental exposure information and genetic risk score and used Cox proportional hazards model to identify individuals at different risk groups for developing of OMPD before the onset of clinical disease. Regarding prediction model and risk factors, predictive methods may vary from study to study. There are some limitations to our study. Misclassification of OPMD in a high-risk group resulted in a very low AUC valueand lower predictive probability of OPMD occurrence **(**S4 Table**)** in OPMD prediction model. Subjects without OPMD may occur to OSCC in the future.

We selected the candidate genes which were reported highly significantly associated with OSCC in male population. We cannot include all possible genes in the prediction model. It resulted in a very low AUC value for GRS. Our study included the small number of OPMD cases which might have resulted in limited statistical power. This study provides evidence of the effects of germ line variants on the development of OPMD. The nature of risk factors for oral malignancy may differ by country and region. After stepwise analysis in selecting risk factors to best fit model in an economically efficient way, OPMDs and OSCC have the same genetic factors and BQ use in the prediction model. The difference is that tobacco smoking is important to OPMD, and alcohol drinking is important to OSCC. The risk factors of OPMD in prediction model needs to be validated in large sample size studies.

## Conclusions

In summary, our study provided evidence that GRS comprised of *FAT1* and *COL9A1* genes was associated with oral malignancy occurrence in male population. For the risk prediction models, combined GRS and environmental factor were highly predicting OSCC occurrence. This prediction model can be applied to identify high-risk subjects with habits of betel quid chewing, cigarette smoking and further provide the appropriate intervention to reduce the risk of oral malignancy occurrence in male population.

## Supporting information

S1 Table Association of genetic variants with the risk of oral cancer. (DOCX)

S2 Table Equation parameters of the OSCC risk prediction model. (DOCX)

S3 Table Equation parameters of the OPMD risk prediction model. (DOCX)

S4 Table Based on the genomic risk score, predictive probability of OPMD occurrence. (DOCX)
