# Supplementary material for: Variants in FAT1 and COL9A1 genes in male population with or without substance use to assess the risk factors for oral malignancy
Source: PLoS One. 2019 Jan 18;14(1):e0210901. doi: 10.1371/journal.pone.0210901 (PMC6338366; doi:10.1371/journal.pone.0210901)
Supplement: S1 Table — (DOCX) [file pone.0210901.s001.docx]

**S1 Table 1. Association of genetic variants with the risk of oral cancer.**

|  |  |  | Minor |  |  |  | Trend^＊^ | Genotyping | Allele |  |
| --- | --- | --- | --- | --- | --- | --- | --- | --- | --- | --- |
| Gene | SNP | N | Allele | MAF | Control | Case | P-value | P-value | P-value | FDR^＆^ |
| TP53 | rs11652704 | 846 | T | 0.13 | 486 | 356 | 0.8619 | 0.7538 | 0.8629 | 0.9313 |
| TP53 | rs12951053 | 846 | C | 0.33 | 481 | 356 | 0.8468 | 0.5231 | 0.8514 | 0.9313 |
| TP53 | rs17882227 | 846 | C | 0.32 | 486 | 356 | 0.8283 | 0.7348 | 0.8334 | 0.9313 |
| CASP8 | rs6745051 | 846 | C | 0.27 | 478 | 356 | 0.3028 | 0.5673 | 0.2886 | 0.9313 |
| CASP8 | rs7608692 | 846 | A | 0.25 | 457 | 343 | 0.7702 | 0.5639 | 0.7829 | 0.9313 |
| CASP8 | rs6754084 | 846 | T | 0.29 | 486 | 356 | 0.9311 | 0.9310 | 0.9313 | 0.9313 |
| FAT1 | rs28647489 | 846 | G | 0.41 | 486 | 360 | 0.0057 | 0.0219 | 0.0047 | 0.0353 |
| FAT1 | rs2306990 | 846 | C | 0.40 | 480 | 355 | 0.8832 | 0.2814 | 0.8838 | 0.9313 |
| FAT1 | rs11724817 | 846 | A | 0.50 | 486 | 357 | 0.9224 | 0.4901 | 0.9201 | 0.9313 |
| FAT1 | rs2130909 | 846 | T | 0.45 | 477 | 356 | 0.5658 | 0.6830 | 0.5542 | 0.9313 |
| FAT1 | rs10009030 | 846 | A | 0.41 | 478 | 353 | 0.3147 | 0.5450 | 0.3262 | 0.9313 |
| FAT1 | rs2637777 | 846 | T | 0.36 | 486 | 356 | 0.5656 | 0.1609 | 0.5660 | 0.9313 |
| FAT1 | rs10434309 | 846 | T | 0.47 | 486 | 356 | 0.3193 | 0.5440 | 0.2989 | 0.9313 |
| COL9A1 | rs550675 | 846 | T | 0.32 | 486 | 360 | <0.0001 | 0.0001 | <0.0001 | 0.0002 |
| NOTCH1 | rs201174576 | 846 | G | 0.35 | 479 | 356 | 0.6921 | 0.7039 | 0.6886 | 0.9313 |

MAF: Minor allele frequency.

^＊^For the additive inheritance model, SNPs were tested for association with oral cancer using the Cochran-Armitage trend test.

^＆^To adjust for the multiple testing, p-value for Cochran-Armitage trend was corrected by false discovery rate correction.
