# Supplementary material for: Variants in FAT1 and COL9A1 genes in male population with or without substance use to assess the risk factors for oral malignancy
Source: PLoS One. 2019 Jan 18;14(1):e0210901. doi: 10.1371/journal.pone.0210901 (PMC6338366; doi:10.1371/journal.pone.0210901)
Supplement: S2 Table — (DOCX) [file pone.0210901.s002.docx]

**S2 Table Equation Parameters of the OSCC risk prediction model.**

| **Variables** | **Remove variables** | **Variables In** | **Model R** | **Estimates** | **F value** | **P- value** |
| --- | --- | --- | --- | --- | --- | --- |
| ***FAT1*** |  | **1** | **0.0153** | **0.58245** | **14.0135** | **0.0043** |
| ***COL9A1*** |  | **2** | **0.015** | **0.37091** | **7.8682** | **0.0045** |
| **BQ Chewing** |  | **3** | **0.4637** | **0.74491** | **40.5662** | **<.0001** |
| **alcohol** |  | **4** | **0.4728** | **0.19206** | **28.8866** | **0.0003** |
| **AGE** |  | **5** | **0.4785** | **0.009284** | **20.2998** | **0.0013** |
| **Smoking** | **Smoking** |  |  |  |  |  |

**Genomic and environmental factor were selected by Stepwise Model. Coefficients obtained from Hazard ratios, a linear equation was constructed to produce OSCC the risk score.** **OSCC risk score=FAT1 risk allele*0.58245+ *COL9A1* risk allele*0.37091+ BQ Chewing*0.74491+ alcohol*0.19206+0.009284 *AGE**
