# Supplementary material for: Variants in FAT1 and COL9A1 genes in male population with or without substance use to assess the risk factors for oral malignancy
Source: PLoS One. 2019 Jan 18;14(1):e0210901. doi: 10.1371/journal.pone.0210901 (PMC6338366; doi:10.1371/journal.pone.0210901)
Supplement: S3 Table — (DOCX) [file pone.0210901.s003.docx]

**S3 Table Equation Parameters of the OPMD risk prediction model.**

| Variables | Remove variables | Variables In | Model R | Estimates | F value | P- value |
| --- | --- | --- | --- | --- | --- | --- |
| GRS |  | 1 | 0.1061 | 3.30404 | 18.04 | <.0001 |
| BQ Chewing |  | 2 | 0.2942 | 1.0129 | 40.24 | <.0001 |
| Smoking |  | 3 | 0.5805 | 1.68576 | 102.41 | <.0001 |
| alcohol | alcohol |  | 0.5869 | 4.1382 | 2.3 | 0.1314 |

**Genomic and environmental factor were selected by Stepwise Model.**

**Coefficients obtained from Hazard ratios,, a linear equation was constructed to produce OPMD the risk score. OPMD risk score=genomic risk score*3.30404+ BQ Chewing*1.0129+ smoking*1.68576.**
