# Supplementary material for: Variants in FAT1 and COL9A1 genes in male population with or without substance use to assess the risk factors for oral malignancy
Source: PLoS One. 2019 Jan 18;14(1):e0210901. doi: 10.1371/journal.pone.0210901 (PMC6338366; doi:10.1371/journal.pone.0210901)
Supplement: S4 Table — (DOCX) [file pone.0210901.s004.docx]

**S4 Table Based on the Genomic risk score, predictive probability of OPMD occurrence.**

| **Disease** | **Genomic risk score** | **Predictive probability of occurrence** |
| --- | --- | --- |
| **OSCC** | **0** | 0.32 |
|  | **1** | 0.44 |
|  | **2** | 0.70 |
| **OPMD** | **0** | 0.04 |
|  | **1** | 0.07 |
|  | **2** | 0.10 |
